# Delegation Opportunities for Malnutrition Care Activities to Dietitian Assistants—Findings of a Multi-Site Survey

**DOI:** 10.3390/healthcare9040446

**Published:** 2021-04-10

**Authors:** Alita Rushton, Adrienne Young, Heather Keller, Judith Bauer, Jack Bell

**Affiliations:** 1 Department of Nutrition and Dietetics, The Prince Charles Hospital, Chermside, QLD 4032, Australia; alita.rushton@health.qld.gov.au; 2 School of Human Movement and Nutrition Sciences, The University of Queensland, St Lucia, QLD 4072, Australia; adrienne.young@health.qld.gov.au (A.Y.); j.bauer1@uq.edu.au (J.B.); 3 Department of Nutrition and Dietetics, Royal Brisbane Women’s Hospital, Herston, QLD 4029, Australia; 4 Schlegel-University of Waterloo Research Institute for Aging, Waterloo, ON N2L 3G1, Canada; hkeller@uwaterloo.ca

**Keywords:** aged, assistant, delegation, diet therapy, dietitian, hospitals, malnutrition, model of care, nutritional support, nutritionists

## Abstract

Approximately one-third of adult inpatients are malnourished with substantial associated healthcare burden. Delegation frameworks facilitate improved nutrition care delivery and high-value healthcare. This study aimed to explore knowledge, attitudes, and practices of dietitians and dietitian assistants regarding delegation of malnutrition care activities. This multi-site study was nested within a nutrition care implementation program, conducted across Queensland (Australia) hospitals. A quantitative questionnaire was conducted across eight sites; 87 dietitians and 37 dietitian assistants responded and descriptive analyses completed. Dietitians felt guidelines to support delegation were inadequate (agreement: <50% for assessment/diagnosis, care coordination, education, and monitoring and evaluation); dietitian assistants perceived knowledge and guidelines to undertake delegated tasks were adequate (agreement: >50% food and nutrient delivery, education, and monitoring and evaluation). Dietitians and dietitian assistants reported confidence to delegate/receive delegation (dietitian agreement: >50% across all care components; dietitian assistant agreement: >50% for assessment/diagnosis, food and nutrient delivery, education, monitoring and evaluation). Practice of select nutrition care activities were routinely performed by dietitians, rather than assistants (*p* < 0.001 across all nutrition care components). The process for care delegation needs to be improved. Clarity around barriers and enablers to delegation of care prior to implementing reforms to the current models of care is key.

## 1. Introduction

Protein-energy malnutrition (malnutrition) is prevalent occurring in approximately one-third of adult inpatients [1,2,3]. Malnutrition results from inadequate macronutrient intake and/or uptake, or when patients have increased nutritional requirements associated with disease or other complications [3]. Impaired food intake or assimilation translates into malnutrition when it adversely impacts the patient’s outcomes, including function, recovery, mortality, length of stay, and hospital costs [2,4].

A high proportion of dietitians both within Australia and internationally work in the hospital setting with a focus on malnutrition care [5,6,7,8,9]. Nutrition screening tools are used to identify patients at risk of malnutrition, who are then referred to a dietitian to enable nutrition assessment and intervention in those identified as at risk [6,10,11]. There has been a marked increase in the proportion of patients screened at risk with the introduction of electronic medical record systems [12]. It has been highlighted that dietitians providing nutrition care for all patients identified as “at risk” of malnutrition is highly individualised and resource intensive, and consequently is unsustainable in managing increased referrals [13]. Current national and international research and guidelines are beginning to explore and advocate for culture change in nutrition care; however, many services are still focused on nutrition care processes reliant on dietitians [13,14,15,16].

The consequent heightened workforce demands and burgeoning healthcare costs, together with directives for workforce reform, highlight the need for a shift in malnutrition care in hospital settings [12,17,18,19]. Moving towards interdisciplinary and/or delegated nutrition care frameworks within inpatient settings has demonstrated improved delivery of nutrition care and patient outcomes, and further support calls to deliver high value healthcare [7,13,14].

The Nutrition Care Process (NCP) outlines the elements that should be considered to deliver high-value nutrition care [6]. Applying the NCP to malnutrition care ensures patients receive a nutrition assessment and diagnosis which inform appropriate nutrition interventions followed by monitoring and evaluation of patient’s nutrition status [6,20]. A recent study has demonstrated that the majority of patients are considered appropriate to receive systematised and/or interdisciplinary nutrition care processes, enabling dietitians or medical nutrition specialists to focus on specialised care for those individuals who are resistant to standard or supportive care approaches [13].

Many dietetic services both within Australia and internationally have access to dietitian assistants (nutrition assistant, dietetic assistants, allied health assistants, dietetic technicians, etc.) [21]. However, there are diverse and sometimes conflicting governance requirements, guidelines, policies and workplace instructions from both professional associations and health services that support or hinder opportunities for delegation of components of NCP activities to assistants [18,21,22,23,24,25].

The extent to which the nutrition and dietetic profession are utilising assistants, and further, which elements of the NCP are routinely delegated is unclear. The underlying knowledge and attitudes that influence dietitian delegation practices to dietitian assistants, for patients with, or at risk of, malnutrition also remain unclear.

Consequently, the aim of this study was to explore knowledge, attitudes, and practices of dietitians and dietitian assistants with respect to delegation of nutrition care activities to assistant staff for patients with, or at risk of, malnutrition.

## 2. Materials and Methods

This is a multi-site study, nested within a multi-phase nutrition care implementation program for patients with, or at risk of, malnutrition [13], conducted across diverse hospitals in Queensland, Australia between December 2018 and June 2019. The study used a quantitative multi-site survey that was tailored from the Malnutrition Knowledge, Attitudes and Practices survey from Canada [26] in consideration of elements from the Allied Health Assistant Framework and NCP [6,18]. Two variations of the questionnaire were developed, one for dietitians (Supplementary 1) and one for dietitian assistants (Supplementary 2). Each questionnaire consisted of 12 questions related to knowledge, attitudes and practices regarding delegation of nutrition care activities with 4- or 5-point Likert scale responses, as well as five demographic questions (age, gender, employment contract: full-time, part-time, casual; number of years practicing, primary unit of work). Face validity was provided from experts in the field, both internal (AY, HK, JJB) and external to the study team (3 dietitian assistant team leaders, 1 state-wide foodservice director, 1 dietitian assistant, and 1 implementation scientist), with consensus achieved for the final questions and format. The surveys were undertaken as part of a state-wide malnutrition care implementation program (SIMPLE II), which aimed to embed the Systematised, Interdisciplinary Malnutrition Program for impLementation and Evaluation (SIMPLE) [13] into routine clinical practice. The study protocol was approved as a low-negligible risk study by the relevant Human Research Ethics Committee (project ID 47929). Participants were recruited from a convenience sample consisting of the eight sites with diverse dietetic and assistant workforce structures and delegation processes who agreed to implement SIMPLE II (Table 1) [13].

The questionnaires were distributed, via email or face-to-face paper copies, to dietetic service managers or team leaders across the selected sites, who then distributed the surveys to their dietetic staff via email and/or staff meetings. Completed questionnaires were returned to AR and JJB, and data were entered by AR into a Microsoft Excel 365 and 2016 (Microsoft Corporation, Redmond, Washington, DC, USA) database. Data analysis was undertaken in Microsoft Excel 365 and 2016 (Microsoft Corporation, Redmond, Washington, DC, USA) and SPSS^®^ (versions 23 and 25) software. Questions that specifically align to the NCP were analysed into the following NCP categories: assessment and diagnosis, food and nutrient delivery, education, care coordination, and monitoring and evaluation. Likert scale responses for each question were assigned a value (0 = strong disagree, 1 = disagree, 2 = neutral, 3 = agree, 4 = strongly agree; 0 = never, 1 = sometimes, 2 = often, 3 = always) to enable comparison of means (using independent t-test) or medians (using Mann–Whitney U test) depending on normality. Responses were then analysed descriptively (frequencies for each response category), with comparisons made between dietitian and dietitian assistant responses using the non-parametric Mann–Whitney U-test, with significance level of *p* < 0.05.

## 3. Results

Completed surveys were returned from all eight sites, with a response rate of 75% (87/116) for dietitians and 90% (37/41) for dietitian assistants. The demographic details of dietitians showed a range across years of practice and work units (Table 2). There was a high predominance towards female gender and age less than 50 years, in line with the profession’s demographics. Representation was focused on full time staff rather than part time or casual staff. Similar characteristics were observed for the dietitian assistant cohort, with the exception of a higher proportion of part time workers and those aged 50 years and older.

Table 3 provides a summary comparison of the median Likert scale responses between the dietitian and dietitian assistant respondents.

### 3.1. Current Knowledge to Support Delegation of Malnutrition Care Activities

**Figure 1:**
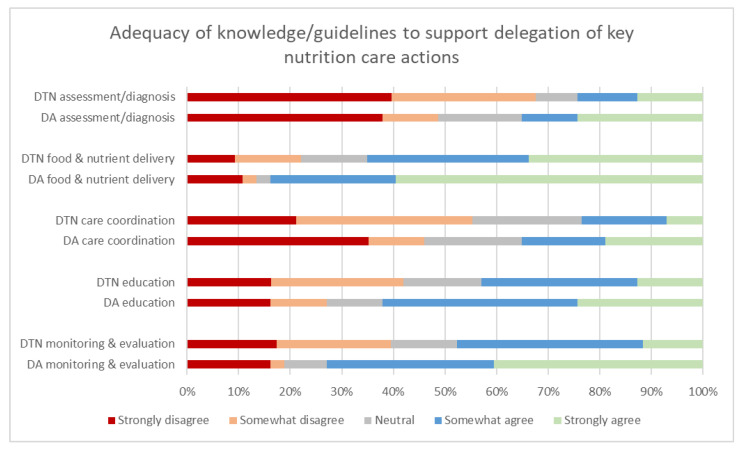
Adequacy of knowledge, guidelines, task instructions and/or tools to support delegation of key nutrition care actions—dietitians’ (DTN) (*n* = 85) * and dietitian assistants’ (DA) (*n* = 37) responses *. * An additional 1 dietitian completed 4 of the 5 question sub-components.

Figure 1 summarises the dietitian versus dietitian assistant responses regarding adequacy of knowledge, guidelines, task instructions and/or tools for various nutrition care actions. The majority of dietitians reported that guidelines were inadequate to support delegation of most nutrition care components to dietitian assistants, with the exception of food and nutrient delivery. However, more than half of dietitian assistants agreed that there was adequate knowledge or guidelines to support delegated actions for patient education and monitoring/evaluation actions, as well as food and nutrient delivery, although only the latter two were significantly different between dietitian and assistant groups (Table 3).

Figure 1 illustrates respondents’ agreement with the question “I currently have adequate guidelines/task instructions/tools to support delegating the following assessments and/or treatments for patients” (dietitians) and “I currently have adequate knowledge or guidelines/task instructions/tools to provide the following delegated assessments and/or treatments for patients” (dietitian assistants). DTN: dietitian, DA: dietitian assistant.

### 3.2. Current Attitudes Surrounding Delegation of Malnutrition Care Activities

**Figure 2:**
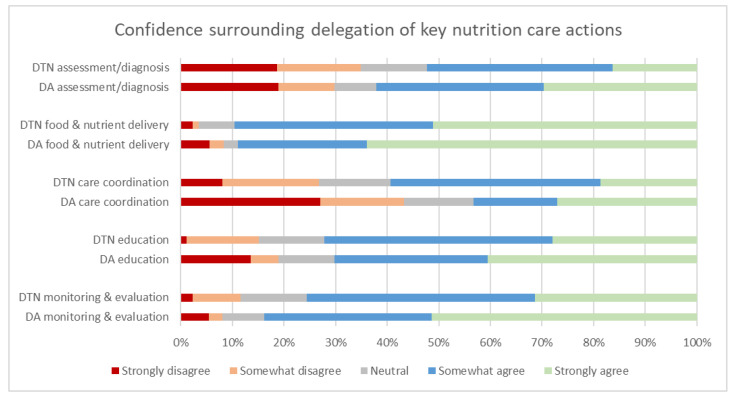
Confidence of dietitians (DTN) (*n* = 86) * and dietitian assistants (DA) (*n* = 36) * surrounding delegation of key nutrition care actions. * An additional 1 dietitian assistant completed 4 of the 5 question sub-components.

Overall, the majority of both dietitians and dietitian assistants reported confidence in delegating, or receiving delegated actions, for most NCP components (Figure 2). There were no differences between dietitian and dietitian assistant workforce confidence responses across nutrition care process actions (Table 3). The dietitian and dietitian assistant workforce both reported the highest level of confidence with delegating food and nutrient delivery actions. Dietitian confidence to delegate actions was lowest for malnutrition assessment and diagnostic care, whilst dietitian assistants were least confident in care coordination (Figure 2). In the dietitian’s survey the question was asked if appropriate resources and staff were available would they be confident to delegate one or more malnutrition care activities to each workforce: AHAs, students, nursing, non-dietetic allied health professional and medical staff”; 98% of dietitians responded positively regarding confidence in delegation to AHAs (with 1% neutral, and 1% not responding) (Supplementary 1, question 6).

Figure 2 illustrates respondents’ agreement with the question “I feel confident to coordinate any interdisciplinary team members (Nurses, Doctors, Allied Health staff, AHA staff) to assist undertaking the following assessments and/or treatments for patients at risk of malnutrition/or who are malnourished” (dietitians) and “If delegated, I would feel confident to provide the following assessments and/or treatments for patients at risk of malnutrition/or who are malnourished.” (dietitian assistants). Complete data was available for 86 dietitians and 36 dietitian assistant staff. DTN: dietitian, DA: dietitian assistant.

### 3.3. Current Practice of Malnutrition Care Activities

**Figure 3:**
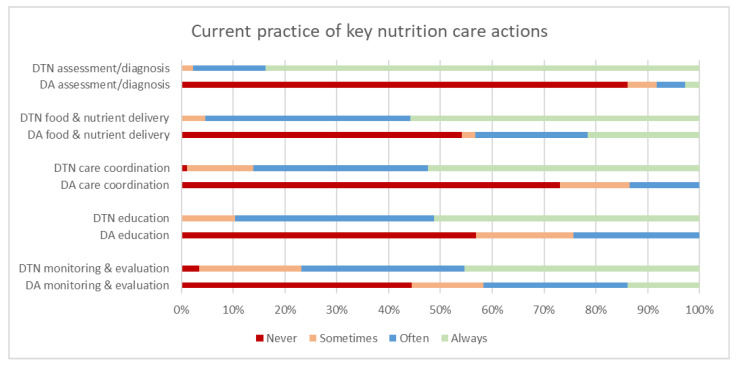
Current practice of key nutrition care actions by dietitians’ (DTN) (*n* = 86) and dietitian assistants’ (DA) (*n* = 35) *. * An additional two dietitian assistants completed 4 of the 5 question sub-components.

Figure 3 highlights that nutrition care activities are routinely performed by dietitians, rather than dietitian assistant staff across sites and across all domains of the nutrition care process; these findings are statistically significant (Table 3). Components most frequently undertaken by dietitian assistants were food and nutrient delivery (43% often or always undertaking this task) and monitoring and evaluation (40% often or always undertaking this task).

Figure 3 illustrates respondents’ agreement with the question “For patients malnourished or at risk of malnutrition that you are involved with on your ward, how often do you individually deliver any of the following assessments and/or treatments?” (dietitians) and “In your current practice, do you individually deliver any of the following assessments and/or treatments for patients at risk of malnutrition/or who are malnourished?” (dietitian assistants). DTN: dietitian, DA: dietitian assistant.

When dietitians were asked if they had enough time to provide individualised malnutrition care for all patients at risk of malnutrition/or who are malnourished admitted to their ward(s), and to complete all other tasks and activities, 75% (*n* = 65) indicated they did not [7% (*n* = 6) neutral response, 17% (*n* = 15) felt they did; *n* = 1 did not respond].

**Figure 4:**
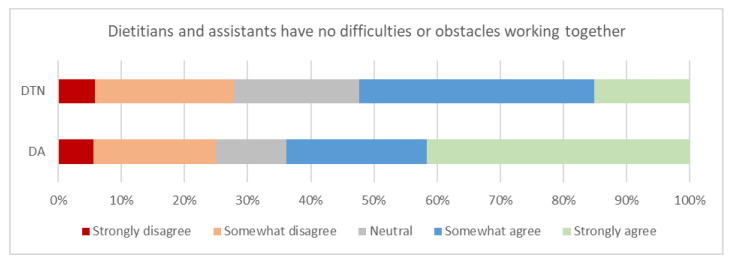
Dietitians’ (DTN) (*n* = 86) * and dietitian assistants’ (DA) (*n* = 36) * opinion regarding no difficulties or obstacles working together. * 1 dietitian and 1 dietitian assistant did not respond to this question.

The majority of dietitian assistants somewhat or strongly agreed that there were no difficulties or obstacles working together with dietitians (62%). Whilst approximately half of dietitians were either neutral or somewhat or strongly disagreed (47%) with this statement, the observed difference was not statistically significant (Figure 4; Table 3).

Figure 4 illustrates respondents’ agreement with the statement “Dietitians and assistants have no difficulties or obstacles to working together to provide malnutrition care. DTN: dietitian, DA: dietitian assistant.

**Figure 5:**
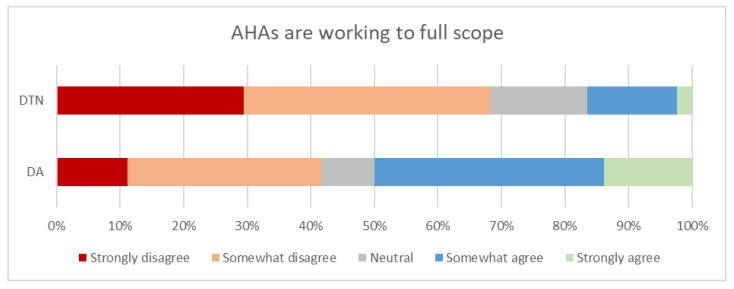
Dietitians’ (DTN) (*n* = 85) * and dietitian assistants’ (DA) (*n* = 36) * perception of dietitian assistants currently working to full scope. * Two dietitians and one dietitian assistant did not respond to this question.

When dietitian assistants were asked if they were currently working to full scope in their role to provide malnutrition care, a split in perception was evident, with a statistically significant difference between dietitian response and dietitian assistant response (Table 3). Half of the dietitian assistant workforce indicated that they strongly or somewhat agreed that they were working to full scope, whilst 40% disagreed or strongly disagreed, and 8% responded as neutral to this statement. However, only 16% of the dietitians strongly agreed or agreed that dietitian assistant staff were being used to full scope in their role to provide malnutrition care, with 67% disagreeing or strongly disagreeing with this statement and 15% responded as neutral (Figure 5).

Figure 5 illustrates respondents’ agreement with the question “In your opinion, are Assistant staff being used to full scope in their role to provide malnutrition care?” (dietitians) and “I feel I am currently working to full scope in my role as an assistant to provide malnutrition care” (dietitian assistants). DTN: dietitian, DA: dietitian assistant

## 4. Discussion

To the authors knowledge, this manuscript is the first to highlight knowledge, attitudes and practices of dietitians and dietitian assistants towards delegation of components of the nutrition care process to dietitian assistant staff.

### 4.1. Delegation Is Not Happening, but Why?

Three-quarters of dietitians indicated that they did not have sufficient time to perform all required care practices for malnourished patients in their hospital. However, there is a discrepancy between perceived dietitian confidence and current delegation practices. The majority of dietitian respondents reported confidence to delegate most nutrition care activities to the dietitian assistant workforce, however assistants are working below their full scope of practice. Results also highlight the poor uptake of dietitian delegation of tasks across most domains of the NCP, with dietitians predominantly undertaking nutrition care across all domains of the NCP (with the exception of food and nutrient delivery). These results are consistent with previous findings that demonstrate nutrition care processes are predominately still being undertaken by dietitians [13].

Some potential reasons for this gap between confidence and practice were identified in this study. Half of dietitians identified potential challenges working with assistants, although the nature of this challenge is unknown. Further, guidance on what to delegate and how to do so appears to be lacking across all NCP domains. Only a minority of dietitians reported adequate knowledge, guidelines, task instructions and/or tools to support delegation of key nutrition care components to assistants. The exceptions were food and nutrient delivery, education, and monitoring and evaluation components of nutrition care, which the majority of the dietitian assistants reported adequate knowledge/guidelines for the delegated process. These activities can be readily standardized processes, requiring minimal individualization for each malnourished patient and processes can be readily devised and followed. As there are local, national, and international guidelines, procedures, task instructions or strategies [18,21,22,25,27] supporting delegation to the assistant workforce, these should be further utilised and developed for implementation and use in hospitals. Our study highlights an urgent need to understand how to increase uptake of these supporting resources to promote translating delegation processes into practice.

### 4.2. Confidence and Knowledge as Precursors to Practice Change

Using a knowledge, attitudes and practice approach has been shown to be useful to change and develop nutrition care processes [26]. The nutrition care components that were the most commonly undertaken individually by the dietitian, and least undertaken by the dietitian assistants, was assessment/diagnosis. This component was also perceived to have the least adequate knowledge/guidelines and low confidence regarding delegation of assessment/diagnosis by both dietitians and dietitian assistants. However, a state-wide clinical task instruction for delegation of a malnutrition assessment tool is available [28] and a small study suggests that allied health assistants in a rural setting were able to apply this clinical task instruction confidently and competently to complete malnutrition assessments with dietitian countersignature [29]. In line with coding guidelines, this clinical task instruction still requires countersignature by dietitian or documentation by medical officer to enable diagnostic coding for malnutrition and case-based reimbursement where applicable. Understanding potential barriers and/or enablers to dietitian assistant supported assessment with dietitian and/or medical officer countersigned diagnosis is an area for further investigation. Implementation models and theories highlight the importance of identifying factors at all levels that will affect the implementation of the desired practice change [30,31,32,33,34]. Implementation of new delegation processes is a complex matter, and the barriers and enablers to shifting from confidence and knowledge to practice require exploration.

Similarly, the care coordination component of nutrition care was thought to be lacking in adequate guidelines for delegation, with low levels of confidence, and less frequency of practice by both dietitians and dietitian assistants. The ambiguity of what tasks could be included in “care coordination” regarding patient’s nutrition care may have contributed to these responses. Further exploration of what activities are involved in care coordination and opportunities for delegation is needed to fully understand the dietetic workforce perceptions concerning this activity. Care coordination may also be linked to the assessment and diagnosis component of care. Part of a comprehensive dietitian assessment includes understanding the factors that impact food and fluid intake and how these can be managed in the disease course [35]. Care coordination is a treatment process that is often tailored to the determinants of eating behaviour; this may explain why this care activity is similarly viewed as less likely to be delegated by dietitians and assistants if it is a process linked to the assessment of malnourished patients.

The food and nutrient delivery component was limited to prescription of high protein, high energy diet and/or mid-meals, not the more specialised activities such as enteral or parental tube feeding. Food and nutrient delivery was one of the highest care activities performed by dietitian assistants and perceived to have the most adequate knowledge/guidelines and highest confidence levels surrounding this task. Interestingly, this component was also the second highest task individually undertaken by dietitians after diagnosis and assessment (Figure 3). This raises a question around current practice of nutrition care. Should both the dietitian assistants and dietitians undertake food and nutrient delivery? Is this high-value healthcare? In the current climate of efficient and effective nutrition care it is essential that patients receive high value healthcare [17]. Prior research has shown that using the multi-disciplinary team to initiate HPHE diets and mid-meals improved patient protein and energy intakes [36,37]. Implementing broader multi-disciplinary team systems and processes that involve some elements of nutrition care, particularly supportive food and nutrient delivery, will ensure patients are still receiving the required nutrition care, while dietitians are reserved for specialised tasks, whether related to food and nutrient delivery or other specialised nutrition care components.

Skill sharing is an emerging concept that has been suggested to facilitate efficient and effective healthcare. Nursing staff who are at the forefront of patient care [38] are especially equipped to provide key nutrition care activities. For example, nutrition risk screening and consequent referral by nursing staff to the dietitian service [39] has been shown to be implementable and improved practice. This step may be an untapped opportunity for nursing staff or the broader multidisciplinary team to take on other nutrition care strategies that do not require specialised dietetic input [13,37,40]. Interestingly, results from this survey highlighted that dietitians were more confident to delegate to dietitian assistants, rather than students, nursing, non-dietetic allied health professionals or the medical team. The gap in understanding as to why there is a lack of confidence in the broader multidisciplinary team requires further exploration.

### 4.3. Is the Dietetic Workforce Ready for Delegation?

Recent findings have indicated that in Queensland hospitals there is a maximum ratio of less than one dietitian assistant to five dietitians (unpublished data). This raises the question regarding whether there are enough dietitian assistants to delegate nutrition care to. The demographics of participants showed a higher prevalence of females for both dietitians and dietitian assistants. However, a more rudimentary consideration is age and experience. The demographics in this paper demonstrated a younger, less experienced dietitian workforce (>75% under 40 years of age; 30% with >10 years’ experience) and an older, more experienced dietitian assistant workforce (69% over 40 years of age; 43% with >10 years’ experience). The findings around confidence in delegation and types of care dietitians think are appropriate for delegation are likely reflective of 70% of dietitians, in this study, having <10 years of experience. Delegation takes not only guidance and support to do, but also confidence in one’s role and how it relates to others in the team. This comes with experience and recognition of the unique contributions of the dietitian in the multidisciplinary team, while sharing in the interdisciplinarity of nutrition care for malnourished patients. Lack of confidence in this unique role may have played into some of the findings in this study. Conversely, the positive confidence levels that were found for some nutrition care components may suggest some readiness of the dietetic workforce for delegation.

Implementation of changes to nutrition care in a hospital setting can be challenging, however, five key areas to support productive change have been identified [16]. One of these key areas is to “involve the relevant people in the change process”; ensuring dietitians, dietitian assistants, managers and broader healthcare team members are involved in the process of exploring and implementing delegation opportunities across the NCP will be vital to successful uptake and sustainability of changes to the nutrition care provided by the workforce [16,33]. Of concern were the views that the working relationship between dietitians and assistants was challenging (Figure 4). Understanding the enablers of these challenges will be important before delegated practices can be expanded.

The dietitian assistant workforce demonstrated polarisation regarding whether they are working to full scope or not, with only 51% feeling they were working to full scope. Addressing this polarisation among the dietitian assistant workforce when exploring delegation opportunities will be helpful in facilitating successful changes in practice. Use of theoretical frameworks and recent research on implementation of nutrition care changes are valuable tools when considering implementation of changes to delegation practices [16,30,31,32,33,34]. Understanding workforce characteristics and the associated challenges and benefits to implementation of tasks and process changes is vital. Additionally, appropriate clinical governance and frameworks surrounding delegation processes will facilitate safe and effective delegation and support the workforce [18,21,22,25].

### 4.4. Implication for Future Practice and Research

Exploring and addressing barriers and enablers surrounding uptake of delegation practices across nutrition care components, will be crucial to further support the readiness of the dietetic workforce to increase delegation and enable both dietitians and dietitian assistants to work to full scope. This will be in many settings a complex change process and we would recommend applying theoretical frameworks, models and governing infrastructure to facilitate safe, effective and sustainable change [16,18,21,22,25,30,31,32,33,34].

Specifically, there are some obvious gaps between dietitian and dietitian assistant knowledge, attitudes and practice that have been highlighted. French et al. describe a useful four step process for developing implementation interventions to change behaviour in a clinical setting: (1) who needs to do what differently? (2) using a theoretical framework which barriers and enablers need to be addressed? (3) which intervention components (behaviour change techniques and mode(s) of delivery) could overcome the modifiable barriers and enhance the enablers? (4) how can behaviour change be measured and understood? [41]. Applying such processes will assist in identifying and addressing the disparities between knowledge, attitudes, and practices.

Workforce culture and team relationships is known to affect changes in the healthcare environment both positively and negatively [15,16,36,42,43]. Further exploratory work to consider the culture and relationships within the dietetic workforce and the broader healthcare team will be useful when planning, implementing, and evaluating changes to delegation processes.

### 4.5. Strengths and Limitations

Participants were from diverse hospitals in Queensland with varying facility size, resources available, and patient populations. This survey also demonstrated a high completion rate (75% dietitians; 90% dietitian assistants). Whilst the results provide novel findings, it is acknowledged that this study has some limitations. The quantitative nature of the questionnaire provided no opportunity for staff to further explain their responses. Additionally, respondents’ perspectives expressed in the questionnaire is subjective, self-reported, consequently we acknowledge that a discrepancy between actual knowledge and the perception of such knowledge may exist. Detailed demographic data regarding dietetic workforce structure, tasks, and associated instruction documents for each participating site was not collected. Subset analysis and comparisons across sites could not be undertaken due to some participating sites having a small number of dietitians and dietitian assistants. Additionally, there are numerous specific activities in nutrition care that could be considered for delegation to dietitian assistants (e.g., collection of anthropometric data, food/drink preferences), but the questions in the survey only included key global components based on consultation and other works in the field [6,13]. This study specifically targeted dietitians’ and dietitian assistants’ perspectives regarding delegation of nutrition care components; authors acknowledge that broader consultation and qualitative methods are likely required to fully understand these perspectives and explore those of others in the team (e.g., managers, allied health, nursing).

## 5. Conclusions

Our findings highlight that dietitians report not having enough time to do all the nutrition care tasks required for malnourished patients in their hospital but are not routinely delegating nutrition care components. Survey findings suggest this is due to a lack of confidence, lack of applied guidelines and task instructions, and perceived difficulties working with assistants. Conversely, assistants appear more likely to report adequate knowledge and confidence to perform delegated tasks. There is potential to improve practice and meet all of the care needs of malnourished patients, but the dietitian workforce will need to be ready and willing to delegate care. The next step is to clearly articulate barriers and enablers to delegation of care, especially assessment, diagnosis and coordination of care, prior to implementing reforms to the current models of care in Australian hospitals.

## Figures and Tables

**Table 1:** Demographics of participating sites.

Site	Bed Numbers	Metro/Regional	Number of Dietitians	Number of Dietitian Assistants
1	450–599	Tertiary and satellite centres	44	14
2	600–749	Tertiary	20	5
3	150–299	Regional	8	6
4	450–599	Tertiary	26	10
5	150–299	Regional	9	6
6	150–299	Regional	6	1
7	300–449	Regional	6	0
8	150–299	Regional	4	1

**Table 2:** Demographics of dietitians (*n* = 87) and dietitian assistants (*n* = 37) participants.

Variable	Dietitians % (*n*)	Dietitian Assistants % (*n*)
**Gender**		
Male	11 (10)	3 (1)
Female	89 (77)	97 (36)
**Age group**		
<30 yrs	35.6 (31)	18.9 (7)
30–39 yrs	40.2 (35)	16.2 (6)
40–49 yrs	20.7 (18)	21.6 (8)
50–59 yrs	1.1 (1)	24.3 (9)
60+ yrs	2.3 (2)	18.9 (7)
Employment contract *		
Full time	69 (60)	29.7 (11)
Part time	29.9 (26)	62.2 (23)
Casual	1.1 (1)	5.4 (2)
Number of years practicing **		
<5 yrs	34.4 (30)	32.4 (12)
6–10 yrs	35.6 (31)	24.3 (9)
10+ yrs	29.8 (26)	43.2 (16)
**Primary unit of work**		
Medical	33.3 (29)	10.8 (4)
Surgical	10.3 (9)	0 (0)
Rehabilitation/sub-acute	5.7 (5)	5.4 (2)
All other	17.2 (15)	21.6 (8)
Multiple units selected	33.3 (29)	62.2 (23)

* Tables not completed for 1 dietitian assistant. ** Number of years was collapsed into 3 categories: <5 yrs (combining <2 and 2–5 years), 6–10 years, and 10+ years (combining 11–20, 21–30, and 31+ years).

**Table 3:** A comparison of the median Likert scale score responses between dietitian and dietitian assistant respondents.

	Dietitian Median (IQR)	Dietitian Assistant Median (IQR)	*p*-Value ^a^
Knowledge to support delegation of malnutrition care activities * ^b^			
Ax and diagnosis	1 (2)	2 (4)	0.240
Food and nutrient delivery	3 (2)	4 (1)	0.013
Care coordination	1 (1)	2 (3)	0.679
Education	2 (2)	3 (3)	0.075
Monitoring and evaluation	2 (2)	3 (2)	0.002
Confidence surrounding delegation of malnutrition care activities * ^c^			
Ax and diagnosis	3 (2)	3 (3)	0.231
Food and nutrient delivery	4 (1)	4 (1)	0.301
Care coordination	3 (2)	2 (4)	0.199
Education	3 (2)	3 (2)	0.632
Monitoring and evaluation	3 (1)	4 (1)	0.059
Practice of malnutrition care activities ** ^d^			
Ax and diagnosis	3 (0)	0 (0)	<0.001
Food and nutrient delivery	3 (1)	0 (2)	<0.001
Care coordination	3 (1)	0 (1)	<0.001
Education	3 (1)	0 (1)	<0.001
Monitoring and evaluation	2 (1)	1 (2)	<0.001
No difficulties working together * ^c^	3 (2)	3 (3)	0.051
Assistants are working to full scope * ^e^	1 (2)	2.5 (2)	0.001

^a^ Mann–Whitney u-test. ^b^ n= 85 dietitians, 37 assistants; ^c^ n = 86 dietitians, 36 assistants; ^d^ n = 86 dietitians, 35 assistants; ^e^ n = 85 dietitians, 36 assistants. * 0 = strongly disagree, 1 = disagree, 2 = neutral, 3 = agree, 4 = strongly agree ** 0 = never, 1 = sometimes, 2 = often, 3 = always.

## Data Availability

Please contact the corresponding author regarding data availability.

## Supplementary Materials

The following are available online at https://www.mdpi.com/article/10.3390/healthcare9040446/s1, Supplementary 1. Delegation survey—for dietitians; Supplementary 2. Delegation Survey—for Dietitian Assistants.
