# Supplementary material for: Delegation Opportunities for Malnutrition Care Activities to Dietitian Assistants—Findings of a Multi-Site Survey
Source: Healthcare (Basel). 2021 Apr 10;9(4):446. doi: 10.3390/healthcare9040446 (PMC8068993; doi:10.3390/healthcare9040446)
Supplement: Supplementary file 1 [file healthcare-09-00446-s001.pdf]

Supplementary 1. Delegation survey—for dietitians

The SIMPLE Approach

DELEGATION SURVEY - Dietitian

Abbreviations
DN = Dietitian
AHA = nutrition assistant/dietetic assistant/dietitian assistant/allied health assistant/allied health worker/diet aide
SAM = supplements as medicine/MedPass
HPHE = high protein, high energy

About You

1. Which type of hospital unit(s) do you primarily work in? *(Please check all that apply)*
- ☐ Medical
- ☐ Surgical
- ☐ Rehabilitation/sub-acute
- ☐ All other, please identify____

2. Are you
- ☐ Full time
- ☐ Part time
- ☐ Casual
- ☐ Other (please identify) ____

3. How many years you have been practising: *(TIP: This is overall, not just in your current hospital)*
- ☐ < 2 years
- ☐ 2—5 years
- ☐ 6—10 years
- ☐ 11—20 years
- ☐ 21—30 years
- ☐ 31+ years

4. Which age group do you fall into?
- ☐ <30 years
- ☐ 30—39 years
- ☐ 40—49 years
- ☐ 50—59 years
- ☐ 60+ years

5. What is your self-identified gender?
- ☐ Male
- ☐ Female
- ☐ Other
- ☐ Prefer not to say

Version 2018/10/12

The chosen SIMPLE activities identified for your hospital to implement may include delegation or team delivery of the following tasks. Please answer the following questions regarding your current practice and perceptions around delegation of these tasks:

1. For patients malnourished or at risk of malnutrition that you are involved with on your ward, how often do you individually deliver any of the following assessments and/or treatments?

Malnutrition assessment/diagnosis (completed by DN only)	Never	Sometimes	Often	Always
DN prescribed HPHE diet if required	Never	Sometimes	Often	Always
DN prescribed HPHE mid-meals if required	Never	Sometimes	Often	Always
DN prescribed supplements or SAM if required	Never	Sometimes	Often	Always
DN completed, discharge planning & clinical handover for malnutrition	Never	Sometimes	Often	Always
DN provided malnutrition education to individual patients or groups	Never	Sometimes	Often	Always
DN provided malnutrition professional development for staff	Never	Sometimes	Often	Always
DN completed, malnutrition related audits	Never	Sometimes	Often	Always
DN led malnutrition advocacy (e.g. mealtime champion)	Never	Sometimes	Often	Always
DN malnutrition monitoring & evaluation	Never	Sometimes	Often	Always

2. Do you coordinate any interdisciplinary team members (Nurses/Doctors/Allied Health staff/AHA staff) to assist undertaking the following assessments and/or treatments for patients at risk of malnutrition/or who are malnourished?

Malnutrition assessment/diagnosis (with DN countersignature)	Never	Sometimes	Often	Always
HPHE diet prescription	Never	Sometimes	Often	Always
HPHE mid-meals prescription	Never	Sometimes	Often	Always
Supplements or SAM prescription	Never	Sometimes	Often	Always
Discharge planning & clinical handover for malnutrition	Never	Sometimes	Often	Always
Malnutrition education to individual patients or groups	Never	Sometimes	Often	Always
Malnutrition professional development for staff	Never	Sometimes	Often	Always
Malnutrition related audits	Never	Sometimes	Often	Always
Malnutrition advocacy (e.g. mealtime champion)	Never	Sometimes	Often	Always
Malnutrition monitoring & evaluation	Never	Sometimes	Often	Always

3. **I feel <u>confident to coordinate</u> any interdisciplinary team members (Nurses, Doctors, Allied Health staff, AHA staff) to assist undertaking the following assessments and/or treatments for patients at risk of malnutrition/or who are malnourished:**

Malnutrition assessment/diagnosis (with DN countersignature)	Strongly disagree	Somewhat disagree	Neutral	Somewhat agree	Strongly agree
HPHE diet prescription	Strongly disagree	Somewhat disagree	Neutral	Somewhat agree	Strongly agree
HPHE mid-meals prescription	Strongly disagree	Somewhat disagree	Neutral	Somewhat agree	Strongly agree
Supplements or SAM prescription (medical staff)	Strongly disagree	Somewhat disagree	Neutral	Somewhat agree	Strongly agree
Discharge planning & clinical handover for malnutrition	Strongly disagree	Somewhat disagree	Neutral	Somewhat agree	Strongly agree
Malnutrition education to individual patients or groups	Strongly disagree	Somewhat disagree	Neutral	Somewhat agree	Strongly agree
Malnutrition professional development for staff	Strongly disagree	Somewhat disagree	Neutral	Somewhat agree	Strongly agree
Malnutrition related audits	Strongly disagree	Somewhat disagree	Neutral	Somewhat agree	Strongly agree
Malnutrition advocacy (e.g. mealtime champion)	Strongly disagree	Somewhat disagree	Neutral	Somewhat agree	Strongly agree
Malnutrition monitoring & evaluation	Strongly disagree	Somewhat disagree	Neutral	Somewhat agree	Strongly agree

4. **I currently have adequate guidelines/task instructions/tools to <u>support delegating</u> the following assessments and/or treatments for patients:**

Malnutrition assessment/diagnosis (with DN countersignature)	Strongly disagree	Somewhat disagree	Neutral	Somewhat agree	Strongly agree
HPHE diet prescription	Strongly disagree	Somewhat disagree	Neutral	Somewhat agree	Strongly agree
HPHE mid-meals prescription	Strongly disagree	Somewhat disagree	Neutral	Somewhat agree	Strongly agree
Supplements or SAM prescription (medical staff)	Strongly disagree	Somewhat disagree	Neutral	Somewhat agree	Strongly agree
Discharge planning & clinical handover for malnutrition	Strongly disagree	Somewhat disagree	Neutral	Somewhat agree	Strongly agree
Malnutrition education to individual patients or groups	Strongly disagree	Somewhat disagree	Neutral	Somewhat agree	Strongly agree
Malnutrition professional development for staff	Strongly disagree	Somewhat disagree	Neutral	Somewhat agree	Strongly agree
Malnutrition related audits	Strongly disagree	Somewhat disagree	Neutral	Somewhat agree	Strongly agree
Malnutrition advocacy (e.g. mealtime champion)	Strongly disagree	Somewhat disagree	Neutral	Somewhat agree	Strongly agree
Malnutrition monitoring & evaluation	Strongly disagree	Somewhat disagree	Neutral	Somewhat agree	Strongly agree

5. **I currently delegate malnutrition care activities to the following groups:**

Medical	Never	Sometimes	Often	Always
Nursing	Never	Sometimes	Often	Always
Non-dietetic allied health professional	Never	Sometimes	Often	Always
AHA	Never	Sometimes	Often	Always
Students	Never	Sometimes	Often	Always

6. **If appropriate resources and staff were available I would be confident to delegate one or more malnutrition care activities to:**

Medical	Strongly disagree	Somewhat disagree	Neutral	Somewhat agree	Strongly agree
Nursing	Strongly disagree	Somewhat disagree	Neutral	Somewhat agree	Strongly agree
Non-dietetic allied health professional	Strongly disagree	Somewhat disagree	Neutral	Somewhat agree	Strongly agree
AHA	Strongly disagree	Somewhat disagree	Neutral	Somewhat agree	Strongly agree
Students	Strongly disagree	Somewhat disagree	Neutral	Somewhat agree	Strongly agree

7. **I have enough time to provide individualised malnutrition care for ALL patients at risk of malnutrition/or who are malnourished admitted to my ward(s), and complete all other tasks and activities that I should be doing:**

Strongly disagree | Somewhat disagree | Neutral | Somewhat agree | Strongly agree

8. **Do you currently actively contribute to clinical team meetings, case conferences and/or ward rounds?**

Never | Sometimes | Often | Always

9. **Do you currently actively contribute to team projects and quality assurance/research activities?**

Never | Sometimes | Often | Always

10. **I would support appropriately trained AHA staff to directly liaise with healthcare providers within the immediate team (e.g. Doctors, Nurses, Allied Health staff):**

Strongly disagree | Somewhat disagree | Neutral | Somewhat agree | Strongly agree

11. **In your opinion, are Assistant staff being used to full scope in their role to provide malnutrition care?**

Strongly disagree | Somewhat disagree | Neutral | Somewhat agree | Strongly agree

12. **Dietitians and assistants have no difficulties or obstacles to working together to provide malnutrition care:**

Strongly disagree | Somewhat disagree | Neutral | Somewhat agree | Strongly agree

Survey tailored from the Allied Health Assistant Framework (AHPOQ) available at https://www.health.qld.gov.au/__data/assets/pdf_file/0017/147500/ahaframework.pdf
And the Malnutrition KAP (Keller et al) available at https://www.ncbi.nlm.nih.gov/pubmed/27775604

Version 2018/10/12

Supplementary 2. Delegation Survey—for Dietitian Assistants

The SIMPLE Approach

DELEGATION SURVEY – Assistant Staff

Abbreviations
DN = Dietitian
AHA = nutrition assistant/dietetic assistant/dietitian assistant/allied health assistant/allied health worker/diet aide
SAM = supplements as medicine/MedPass
HPHE = high protein, high energy
At risk of malnutrition = As locally defined for example MST 2+ or below specified BMI cut point or specified high risk patient such as an acute hip fracture patient

About You

1. Which type of hospital unit(s) do you primarily work in? *(Please check all that apply)*

- ☐ Medical
- ☐ Surgical
- ☐ Rehabilitation/sub-acute
- ☐ All other, please identify____

3. Are you

- ☐ Full time
- ☐ Part time
- ☐ Casual
- ☐ Other (please identify) ____

4. How many years you have been practising: *(TIP: This is overall, not just in your current hospital)*

- ☐ < 2 years
- ☐ 2—5 years
- ☐ 6—10 years
- ☐ 11—20 years
- ☐ 21—30 years
- ☐ 31+ years

5. Which age group do you fall into?

- ☐ <30 years
- ☐ 30—39 years
- ☐ 40—49 years
- ☐ 50—59 years
- ☐ 60+ years

6. What is your self-identified gender?

- ☐ Male
- ☐ Female
- ☐ Other
- ☐ Prefer not to say

The chosen SIMPLE activities identified for your hospital include delegation of the following tasks. Please answer the following questions regarding your current practice and perceptions around delegation of these tasks:

1. In your current practice, do you individually deliver any of the following assessments and/or treatments for patients at risk of malnutrition/or who are malnourished?

AHA malnutrition diagnosis (eg SGA/PG-SGA with DN countersignature)	Never	Sometimes	Often	Always
AHA prescribed HPHE diet	Never	Sometimes	Often	Always
AHA prescribed HPHE mid-meals	Never	Sometimes	Often	Always
AHA completed, discharge planning & clinical handover for malnutrition	Never	Sometimes	Often	Always
AHA provided malnutrition education to individual patients or groups	Never	Sometimes	Often	Always
AHA provided malnutrition professional development for staff	Never	Sometimes	Often	Always
AHA completed, malnutrition related audits	Never	Sometimes	Often	Always
AHA malnutrition monitoring & evaluation	Never	Sometimes	Often	Always

2. If delegated, I would feel confident to provide the following assessments and/or treatments for patients at risk of malnutrition/ or who are malnourished

Malnutrition diagnosis (eg SGA/PG-SGA with DN countersignature)	Strongly disagree	Somewhat disagree	Neutral	Somewhat agree	Strongly agree
Prescribe HPHE diet	Strongly disagree	Somewhat disagree	Neutral	Somewhat agree	Strongly agree
Prescribe HPHE mid-meals	Strongly disagree	Somewhat disagree	Neutral	Somewhat agree	Strongly agree
Discharge planning & clinical handover for malnutrition	Strongly disagree	Somewhat disagree	Neutral	Somewhat agree	Strongly agree
Malnutrition education to individual patients or groups	Strongly disagree	Somewhat disagree	Neutral	Somewhat agree	Strongly agree
Malnutrition professional development for staff	Strongly disagree	Somewhat disagree	Neutral	Somewhat agree	Strongly agree
Malnutrition related audits	Strongly disagree	Somewhat disagree	Neutral	Somewhat agree	Strongly agree
Malnutrition advocacy (e.g. mealtime champion)	Strongly disagree	Somewhat disagree	Neutral	Somewhat agree	Strongly agree
Malnutrition monitoring & evaluation	Strongly disagree	Somewhat disagree	Neutral	Somewhat agree	Strongly agree

3. Do you support Dietitians or other staff or students to provide any of the following malnutrition care assessments and/or treatments?

Malnutrition diagnosis (eg SGA/PG-SGA with DN countersignature)	Never	Sometimes	Often	Always
Prescribe HPHE diet	Never	Sometimes	Often	Always
Prescribe HPHE mid-meals	Never	Sometimes	Often	Always
Prescribe supplements or SAM	Never	Sometimes	Often	Always
Discharge planning & clinical handover for malnutrition	Never	Sometimes	Often	Always
Malnutrition education to individual patients or groups	Never	Sometimes	Often	Always
Malnutrition professional development for staff	Never	Sometimes	Often	Always
Malnutrition related audits	Never	Sometimes	Often	Always
Malnutrition monitoring & evaluation	Never	Sometimes	Often	Always

4. I currently have adequate knowledge or guidelines/task instructions/tools to provide the following delegated assessments and/or treatments for patients:

Malnutrition diagnosis (eg SGA/PG-SGA with DN countersignature)	Strongly disagree	Somewhat disagree	Neutral	Somewhat agree	Strongly agree
Prescribe HPHE diet	Strongly disagree	Somewhat disagree	Neutral	Somewhat agree	Strongly agree
Prescribe HPHE mid-meals	Strongly disagree	Somewhat disagree	Neutral	Somewhat agree	Strongly agree
Prescribe supplements or SAM	Strongly disagree	Somewhat disagree	Neutral	Somewhat agree	Strongly agree
Discharge planning & clinical handover for malnutrition	Strongly disagree	Somewhat disagree	Neutral	Somewhat agree	Strongly agree
Malnutrition education to individual patients or groups	Strongly disagree	Somewhat disagree	Neutral	Somewhat agree	Strongly agree
Malnutrition professional development for staff	Strongly disagree	Somewhat disagree	Neutral	Somewhat agree	Strongly agree
Malnutrition related audits	Strongly disagree	Somewhat disagree	Neutral	Somewhat agree	Strongly agree
Malnutrition advocacy (e.g. mealtime champion)	Strongly disagree	Somewhat disagree	Neutral	Somewhat agree	Strongly agree
Malnutrition monitoring & evaluation	Strongly disagree	Somewhat disagree	Neutral	Somewhat agree	Strongly agree

5. **I currently have adequate skills to provide the following delegated assessments and/or treatments for patients:**

Malnutrition diagnosis (eg SGA/PG-SGA with DN countersignature)	Strongly disagree	Somewhat disagree	Neutral	Somewhat agree	Strongly agree
Prescribe HPHE diet	Strongly disagree	Somewhat disagree	Neutral	Somewhat agree	Strongly agree
Prescribe HPHE mid-meals	Strongly disagree	Somewhat disagree	Neutral	Somewhat agree	Strongly agree
Prescribe supplements or SAM	Strongly disagree	Somewhat disagree	Neutral	Somewhat agree	Strongly agree
Discharge planning & clinical handover for malnutrition	Strongly disagree	Somewhat disagree	Neutral	Somewhat agree	Strongly agree
Malnutrition education to individual patients or groups	Strongly disagree	Somewhat disagree	Neutral	Somewhat agree	Strongly agree
Malnutrition professional development for staff	Strongly disagree	Somewhat disagree	Neutral	Somewhat agree	Strongly agree
Malnutrition related audits	Strongly disagree	Somewhat disagree	Neutral	Somewhat agree	Strongly agree
Malnutrition advocacy (e.g. mealtime champion)	Strongly disagree	Somewhat disagree	Neutral	Somewhat agree	Strongly agree
Malnutrition monitoring & evaluation	Strongly disagree	Somewhat disagree	Neutral	Somewhat agree	Strongly agree

6. **Do you currently initiate changes to malnutrition care treatments guided by clinical tools / guidelines / task instructions?**

   Never Sometimes Often Always

7. **If required, I would feel comfortable to initiate changes to malnutrition care treatments guided by clinical tools/guidelines/task instructions:**

   Strongly disagree Somewhat disagree Neutral Somewhat agree Strongly agree

8. **Do you currently actively contribute to clinical team meetings, case conferences and/or ward rounds?**

   Never Sometimes Often Always

9. **Do you currently actively contribute to team projects and quality assurance/research activities?**

   Never Sometimes Often Always

10. **Do you currently refer to/liaise with healthcare providers within the immediate team (e.g. Doctors, Nurses, Allied Health staff)?**

    Never Sometimes Often Always

11. **I feel I am currently working to full scope in my role as an assistant to provide malnutrition care:**

    Strongly disagree Somewhat disagree Neutral Somewhat agree Strongly agree

12. **Dietitians and assistants have no difficulties or obstacles to working together to provide malnutrition care:**

    Strongly disagree Somewhat disagree Neutral Somewhat agree Strongly agree

Survey tailored from the Allied Health Assistant Framework (AHPOQ) available at https://www.health.qld.gov.au/__data/assets/pdf_file/0017/147500/ahaframework.pdf
And the Malnutrition KAP (Keller et al) available at https://www.ncbi.nlm.nih.gov/pubmed/27775604

Version 3 11/10/2018
